# PDCL2 is essential for spermiogenesis and male fertility in mice

**DOI:** 10.1038/s41420-022-01210-2

**Published:** 2022-10-17

**Authors:** Minyan Li, Yuxi Chen, Jianping Ou, Junjiu Huang, Xiya Zhang

**Affiliations:** 1 Center for Reproductive Medicine, the Third Affiliated Hospital, Sun Yat-sen University, Guangzhou, 510630 China; 2 MOE Key Laboratory of Gene Function and Regulation, State Key Laboratory of Biocontrol, School of Life Sciences, Sun Yat-sen University, Guangzhou, 510275 China; 3 Key Laboratory of Reproductive Medicine of Guangdong Province, The First Affiliated Hospital and School of Life Sciences, Sun Yat-sen University, Guangzhou, 510275 China

**Keywords:** Genetic predisposition to disease, Spermatogenesis

## Abstract

Patients with teratozoospermia exhibit low phosducin-like protein (*Pdcl2*) expression. As a member of the phosducin family, chaperonin-related *Pdcl2*, a germline-specific gene, may be involved in germ cell protein folding. Given that PDCL2 is highly conserved in evolution, it may be indispensable for mammalian spermiogenesis; however, the function of PDCL2 in higher mammalian species remains unknown. To determine the role of PDCL2 in male fertility, we generated *Pdcl2* knockout mice using CRISPR/Cas9. Our results revealed that *Pdcl2* heterozygous (*Pdcl2*^*+/−*^) male mice were normal, but male *Pdcl2*-null *(Pdcl2*^*−/−*^) mice were infertile. Accordingly, *Pdcl2*^*−/−*^ male mice exhibited lower testis weight, epididymis weight, and sperm number than *Pdcl2*^*+/+*^ mice. Moreover, *Pdcl2*^*−/−*^ mice displayed malformed and immotile sperm. Apoptotic cells were significantly enhanced in *Pdcl2*^*−/−*^ testes and epididymis when compared with those in wild-type mice. Mechanistically, PDCL2 can interact with the CCT complex, and dysfunction in this complex might lead to infertility in *Pdcl2*^*−/−*^ male mice. Collectively, these findings confirm that *Pdcl2* knockout leads to male infertility in mice and that PDCL2 may function as a chaperone to promote protein folding during spermiogenesis.

## Introduction

According to epidemiological investigations, 8–12% of all couples worldwide suffer from infertility, and male factors contribute to approximately 50% of incidences [1]. Sperm malformation is one of the key factors that leads to male infertility in humans. Interestingly, patients with teratozoospermia exhibit low levels of phosducin-like protein 2 (*Pdcl2*) expression [2]. Chaperonin-related *Pdcl2*, a member of the phosducin family, is a germline-specific gene [3, 4]. The phosducin family members, except phosducin, play a functional role in assisting protein folding [5–20]. *Pdcl2* is highly conserved in evolution [3, 21, 22]. Overexpression of mouse PDCL2 in a deficient yeast strain can completely rescue the meiotic defect phenotype [3], revealing a conserved function from yeast to mammalian cells. However, the role of PDCL2 in higher eukaryotes remains unclear.

Some of the phosducin-like proteins are required for cytosolic chaperonin complex (CCT/TRiC)-mediated protein folding [5, 11, 13–15, 17]. CCT belongs to the group II chaperonins, with its two-stack barrel-like structure, which provides sequestered space for protein folding in an ATP-dependent manner [23, 24]. Although the simultaneous presence of phosducin-like proteins and the CCT complex dramatically enhances the protein folding ability when compared with CCT alone, the underlying regulatory mechanism remains elusive.

Considering the conserved sequence of PDCL2 among species, and the fact that the orthologue of PDCL2 plays a role in protein folding [14–16], PDCL2 may be involved in protein folding in the germline. In the present study, we generated knockout mouse models to investigate the role of PDCL2 in spermatogenesis.

## Results

### *Pdcl2* is predominantly expressed in Testis with a stage-specific pattern

**Fig. 1:**
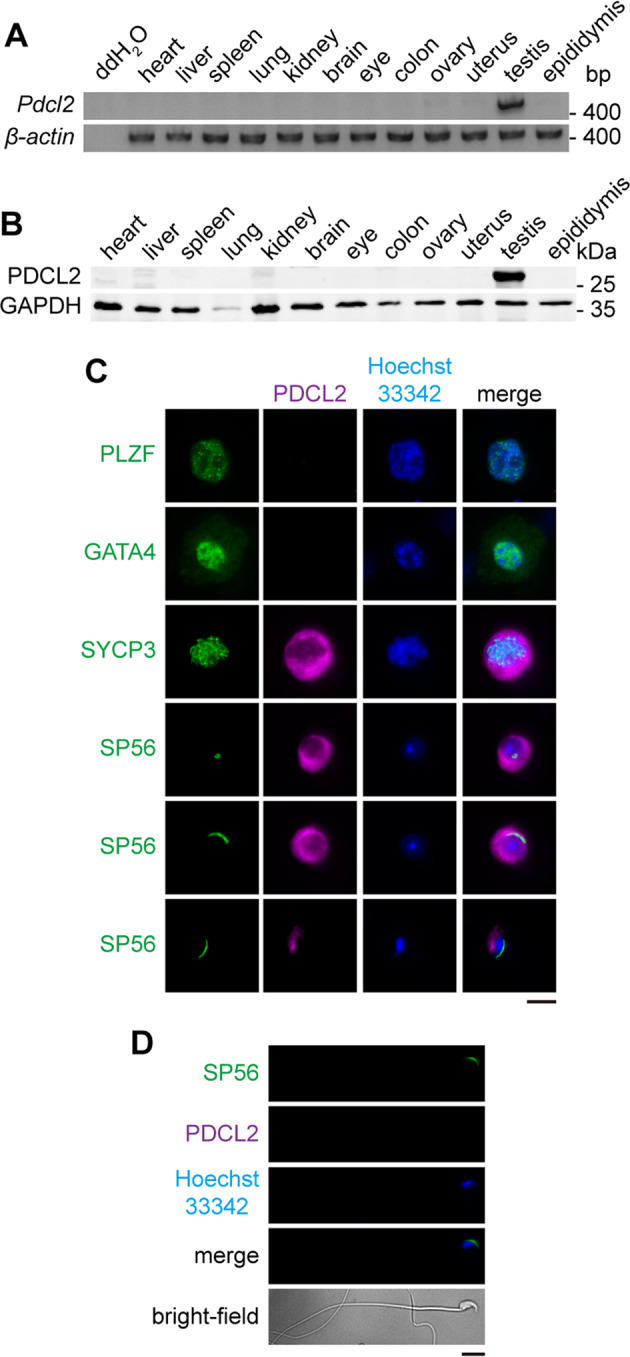
*Pdcl2* is predominantly expressed in testis with a stage-specific pattern. **A**, **B** Expression analysis in various tissues from wild-type 11-week-old C57BL/6 mice. **A** RT-PCR results. The ddH_2_O group was used as PCR control. The *β-actin* group was used as loading control. **B** Western blot analysis. GAPDH was used as loading control. **C**, **D** Immunofluorescence staining of different germ cells extracted from testes and mature sperm isolated from cauda epididymis of wild-type 11-week-old C57BL/6 mice. Green indicates different markers of the cells. PLZF is a marker of spermatogonial stem cell. SYCP3 is a synaptonemal complex component. SP56 is a constituent of the acrosomal matrix. PDCL2 was labeled in magenta. Hoechst 33342 labeled cell nuclear was in blue. Bar, 10 µm.

*Pdcl2*, or named *MgcPhLP*, refers to mouse germ cell-specific phosducin-like protein [3]. Herein, we examined adult mouse tissues to confirm the specific expression of *Pdcl2* in germ cells. Detection of *Pdcl2* mRNA and PDCL2 protein in a series of tissues showed its expression in the testis exclusively (Fig. 1A, B), as determined by microarrays in humans (Fig. S3A) and mice (Fig. S3B).

We performed immunofluorescence staining of germ cells from wild-type mouse testis and observed stage-specific expression patterns of PDCL2 during spermatogenesis. Different biomarkers of germ cells have been used to identify distinct cell types. PLZF-positive cells are undifferentiated, differentiating into spermatogonia in the testis [25, 26]. GATA4-positive cells are somatic cells in testis [27, 28]. SYCP3 is a component of the synaptonemal complex in meiotic cells [29, 30]. SP56 is a component of the acrosomal complex in sperm [31]. PDCL2 were detected in germ cells from meiotic cells to elongated spermatids at all spermatogenesis stages, but not in mitotic cells or spermatozoa isolated from cauda epididymis (Fig. 1C, D). Consistently, *Pdcl2* microarrays of different types of germ cells revealed expression in pachytene spermatocytes and round spermatids but not in spermatogonia stem cells (Fig. S3C).

Overall, these results confirmed that *Pdcl2* is predominantly expressed in male germ cells at a specific stage, indicating that *Pdcl2* might have a potential function in spermatogenesis.

### Generation of *Pdcl2* Knock-out Mice

**Fig. 2:**
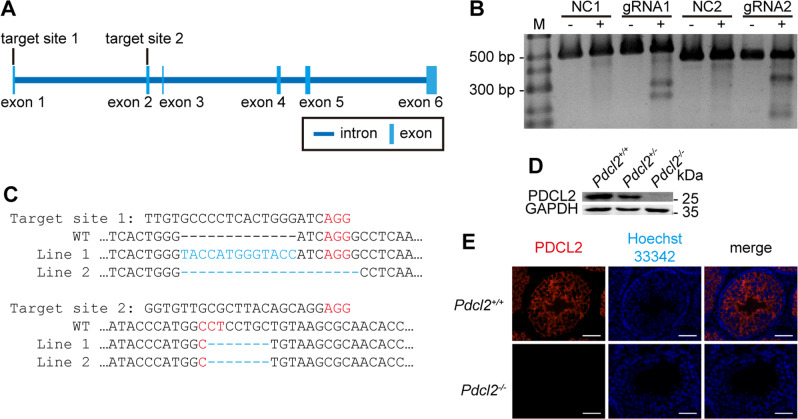
Generation of *Pdcl2* knock-out mice using CRISPR/Cas9. **A** Schematic representation of the gRNA target sites of the *Pdcl2* gene. Exon 1 is the noncoding region. Exon 2 is the beginning of the coding region. **B** Efficacy of CRISPR/Cas9 with the two designated gRNA. V6.5 mouse embryonic stem cells were transfected with different gRNA-containing pX330 or GFP plasmid as a negative control. Targeted genomic region was PCR amplified and then digested by T7 Endonuclease I. M, molecular weight markers. NC, negative control. **C** Genotype of the *Pdcl2* knock-out mice. Both gRNAs were used simultaneously, generating indels in both gRNA target sites in the *Pdcl2* knock-out mice. Indels were generated as highlighted in blue. Protospacer adjacent motifs (PAM) were labeled in red. Minus signs were used to fill in the blanks of the alignment. Apostrophes replaced the omitted sequences due to the demonstration clarity purpose. **D**, **E** Confirmation of PDCL2 deficiency in *Pdcl2*^*−/−*^ mice. **D** Representative western blot analysis of testes lysate. GAPDH was used as loading control. **E** Representative immunofluorescent analysis of testes sections. Mouse testes were paraffin-embedded and sectioned in 7 µm. PDCL2 was labeled in red. Hoechst 33342 labeled cell nuclear was in blue. Bar, 50 µm.

As PDCL2 protein exists in cell types ranging from meiotic cells to elongated spermatids, we disrupted the *Pdcl2* gene to examine its role in germ cell development in vivo. We constructed *Pdcl2* knockout mice using CRISPR/Cas9 system. Two gRNAs targeting exon 1 and exon 2 of *Pdcl2* (Fig. 2A) were designed using http://crispr.mit.edu. Plasmids expressing Cas9 and gRNAs were transfected into mouse V6.5 embryonic stem cells (ESCs). The genome editing efficiency was determined using the T7E1 assay. Both gRNAs showed notable cleavage of the target site in mouse ESCs (Fig. 2B). Cas9 mRNA and gRNAs were injected into the cytoplasm of mouse zygotes to produce mutant mice. First-generation mutant mice were mated with wild-type C57BL/6 mice for three generations to eliminate off-target effects of the CRISPR/Cas9 system. Fourth-generation of *Pdcl2*^*+/−*^ mice were mated to generate *Pdcl2*^*+/+*^, *Pdcl2*^*+/−*^*,* and *Pdcl2*^*−/−*^ mice.

To verify *Pdcl2* mutations in mice, the offspring genotypes were detected using Sanger sequencing. Frame-shift mutations were generated at both gRNA target sites (Fig. 2C). We performed western blot analysis of testis lysates with PDCL2 polyclonal antibody, which revealed PDCL2 expression in *Pdcl2*^*+/+*^ and *Pdcl2*^*+/−*^ mice, but not in *Pdcl2*^*−/−*^mice (Fig. 2D). As expected, PDCL2 expression was not detected in the seminiferous tubules of *Pdcl2*^*−/−*^ mice, as shown by immunostaining (Fig. 2E). These results indicated the successful knockout of *Pdcl2* by CRISPR/Cas9.

### Male *Pdcl2*^*−/−*^ Mice Were Infertile with Abnormal Spermatogenesis

**Fig. 3:**
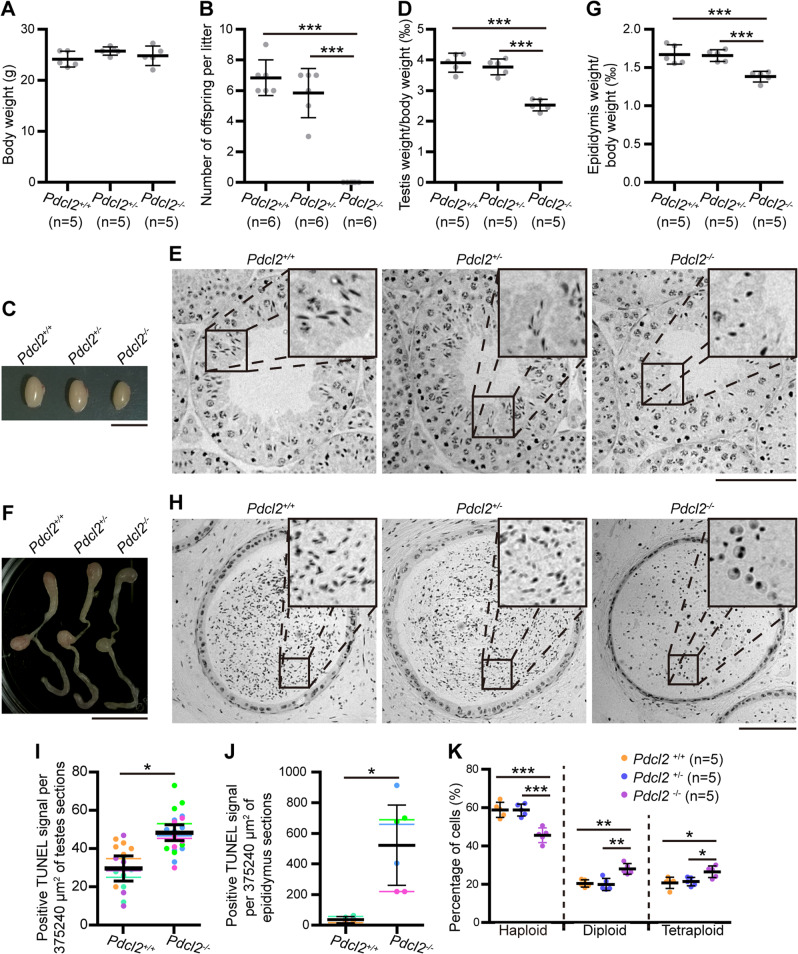
*Pdcl2* is required for spermiogenesis and fertility of male mice. **A** Body weight of 11-week-old male mice. **B** Fertility test. *Pdcl2*^*+/+*^, *Pdcl2*^*+/−*^*,* and *Pdcl2*^*−/−*^ males were mated with wild-type C57BL/6 female mice for one month period of continuous mating. Six adult male mice of each genotype were used. *Pdcl2*^*−/−*^ males gave birth to no pups. **C**, **F** Appearance of testis or epididymis. Bar, 1 cm. **D**, **G** Reduced testis and epididymis weight of *Pdcl2*^*−/−*^ mice. The tissue to body ratio was used to normalize the data. Each sample dot represents the average parameter of both sides of testis or epididymis from the same mouse. **E**, **H** Hematoxylin and eosin stained cross sections of paraffin-embedded mouse testis or cauda epididymis. Bar, 100 µm. **E** Stage XI seminiferous tubule cross section was shown. Less elongated spermatids could be found in *Pdcl2*^*−/−*^ mice. **H** Round shaped cells instead of elongated spermatids could be observed in the epididymis of *Pdcl2*^*−/−*^ mice. **I**, **J** Apoptosis analysis of mouse testis or cauda epididymis section. TUNEL positive signals were counted from pictures of the stained sections. The pictures were taken at random places of the specimens. Each picture covered an area of 375,240 µm^2^. Each dot of the diagram represented a count number of one picture. Different color of dots represented datasets from different specimens. Lines with different color represented means of the datasets. Lines in black represented means of the three means of each group. **K** Percentage of germ cells with different ploidy type in testes of 11-week-old male mice. More than 10^4^ cells of each sample were gated. Data of this figure were represented as mean ± standard deviation. *, *p* < 0.05; **, *p* < 0.01; ***, *p* < 0.001. *n*, mouse number.

*Pdcl2*^*−/−*^ mice were normal in appearance and presented no obvious phenotypic differences in growth when compared with *Pdcl2*^*+/+*^ and *Pdcl2*^*+/−*^ mice (Fig. 3A). However, male *Pdcl2*^*−/−*^ mice failed to produce offspring after mating with normal wild-type female mice following one month of continuous mating (Fig. 3B). Meanwhile, *Pdcl2*^*+/−*^ mice of both sexes and female *Pdcl2*^*−/−*^ mice were fertile.

To determine the underlying mechanims, we dissected the *Pdcl2*^*−/−*^ mice for further examination. *Pdcl2*^*−/−*^ mice exhibited significantly smaller testes than *Pdcl2*^*+/+*^ and *Pdcl2*^*+/−*^ littermates (Fig. 3C). The testes weight of *Pdcl2*^*−/−*^ mice was reduced by approximately 30–40% (testis to body weight ratio of *Pdcl2*^*−/−*^ mice was 2.52 ± 0.19‰ compared to 3.91 ± 0.31‰ of *Pdcl2*^*+/+*^ mice and 3.77 ± 0.26‰ of *Pdcl2*^*+/−*^ mice) at 11 weeks, whereas no difference was observed between testes of *Pdcl2*^*+/+*^ and *Pdcl2*^*+/−*^ mice (Fig. 3D). Hematoxylin and eosin staining of the seminiferous epithelium of *Pdcl2*^*−/−*^ mice revealed histological abnormalities. *Pdcl2*^*−/−*^ mice showed a disruption of spermiogenesis at elongating spermatid stages. We detected markedly few elongated spermatids in stages XI-VIII seminiferous tubules, and most elongated spermatids presented deformities of spermatid nuclei in *Pdcl2*^*−/−*^ mice (Fig. 3E). Compared with *Pdcl2*^*+/+*^ mice, *Pdcl2*^*+/−*^ mouse testes showed no histological abnormalities.

Likewise, *Pdcl2*^*−/−*^ mice displayed abnormalities in the epididymis, which appeared more transparent (Fig. 3F), thereby suggesting less sperm in the epididymis. The epididymis weight of the *Pdcl2*^*−/−*^ mice was reduced by approximately 10-15% (epididymis to body weight ratio of *Pdcl2*^*-/-*^ mice was 1.40 ± 0.03‰ compared with 1.64 ± 0.13‰ in *Pdcl2*^*+/+*^ mice and 1.61 ± 0.06‰ in *Pdcl2*^*+/−*^ mice) (Fig. 3G). Histological analysis of the epididymis showed that *Pdcl2*^*−/−*^ mice had fewer spermatozoa in cauda epididymis and most of the spermatozoa were not elongated while with bigger cell size compared to those of the *Pdcl2*^*+/+*^ and *Pdcl2*^*+/−*^ mice (Fig. 3H).

To determine the underlying cause of weight loss in *Pdcl2*^*−/−*^ mice testes and epididymis, we first examined germ cells apoptosis. Apoptosis plays a key role in spermatogenesis quality control [32]. Dysfunctional spermatogenesis typically relies on apoptosis to eliminate dysfunctional cells. TUNEL assays of *Pdcl2*^*+/+*^ and *Pdcl2*^*−/−*^ testes and epididymis showed increased apoptotic cells in *Pdcl2*^*−/−*^ mice (Fig. 3I, J; Fig. S4), potentially accounting for the decrease in testes and epididymis weight.

The DNA content of germ cells in the testes was analyzed using flow cytometry. Although *Pdcl2*^*+/+*^ and *Pdcl2*^*+/−*^ had a similar proportion of haploid, diploid and tetraploid cells, *Pdcl2*^*−/−*^ presented fewer haploid cells, and hence a greater number of diploid and tetraploid cells (Fig. 3K). No significant difference was found in the ratio of diploid and tetraploid cells (1.00 ± 0.16 in *Pdcl2*^*+/+*^, 0.94 ± 0.22 in *Pdcl2*^*+/−*^, and 1.07 ± 0.17 in *Pdcl2*^*−/−*^), suggesting that spermatogenesis before meiosis MI stage may be normal.

### Oligo-astheno-teratozoospermia of *Pdcl2*^*−/−*^ Mice

**Fig. 4:**
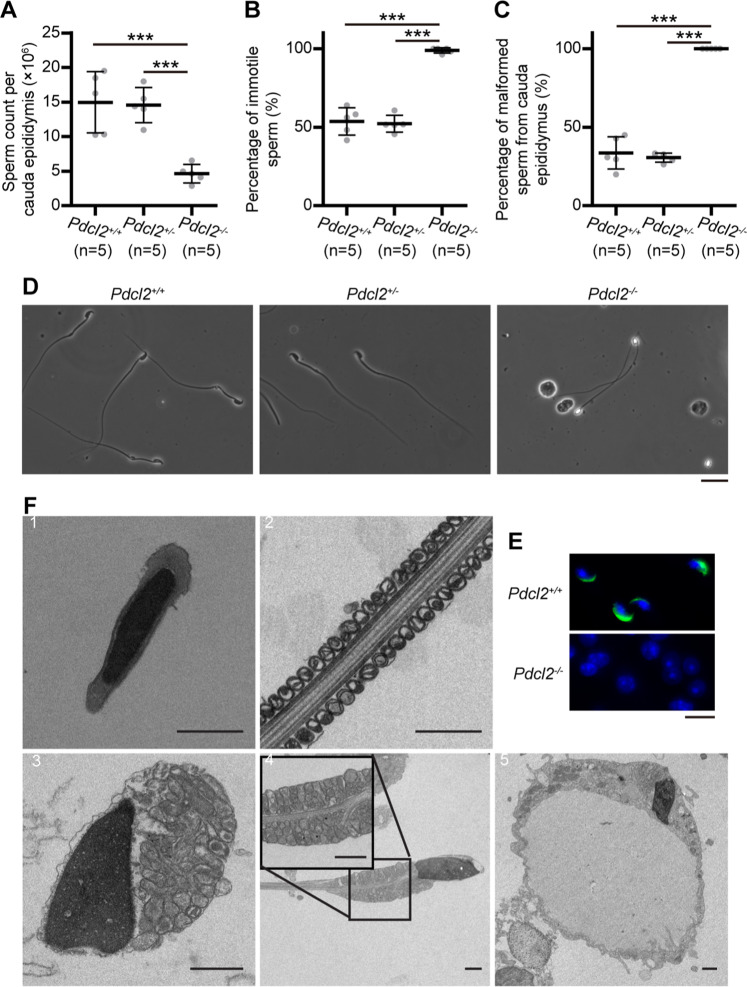
Malformed sperm of *Pdcl2*^*−/−*^ mice. **A** Sperm number per cauda epididymis of 11-week-old male mice. **B** Percentage of immotile sperm. **C** Percentage of malformed sperm. Data of this figure were represented as mean ± standard deviation. *, *p* < 0.05; **, *p* < 0.01; ***, *p* < 0.001. n, mouse number. **D** Light microscopic analysis of sperm extracted from cauda epididymis. Bar, 20 µm. **E** Immunofluorescent analysis of the acrosomal structure of sperm extracted from cauda epididymis. SP56 is a constituent of the acrosomal matrix and was labeled in green. Hoechst 33342 labeled cell nuclear was in blue. Bar, 10 µm. **F** Observation of ultrastructure of sperm with transmission electron microscope. 1, sperm head of wild-type mice. 2, sperm midpiece of wild-type mice. 3–5, sperm of *Pdcl*^*−/−*^ mice, with improper condensed nucleus and abnormal mitochondrial distribution. 3, head and tail were twined together. 4, the axoneme was branched. 5, large vacuole. Bar, 1 µm.

To investigate how PDCL2 deficiency leads to male infertility, we examined sperm squeezed from the cauda epididymis of *Pdcl2*^*+/+*^, *Pdcl2*^*+/−*^, and *Pdcl2*^*−/−*^ mice and quantified the obtained sperm samples. Although we detected no difference in the number of *Pdcl2*^*+/+*^ and *Pdcl2*^*+/−*^ sperm, *Pdcl2*^*−/−*^ sperm were reduced in number by approximately 70% (sperm count of *Pdcl2*^*−/−*^ mice was 4.63 ± 1.34 × 10^6^ compared with 14.96 ± 4.43 × 10^6^ of *Pdcl2*^*+/+*^ mice and 14.57 ± 2.55 × 10^6^ of *Pdcl2*^*+/−*^ mice) (Fig. 4A). Sperm motility was determined by computer-assisted sperm analysis. All the sperm from *Pdcl2*^*−/−*^ mice were immotile (Fig. 4B).

**Fig. 5:**
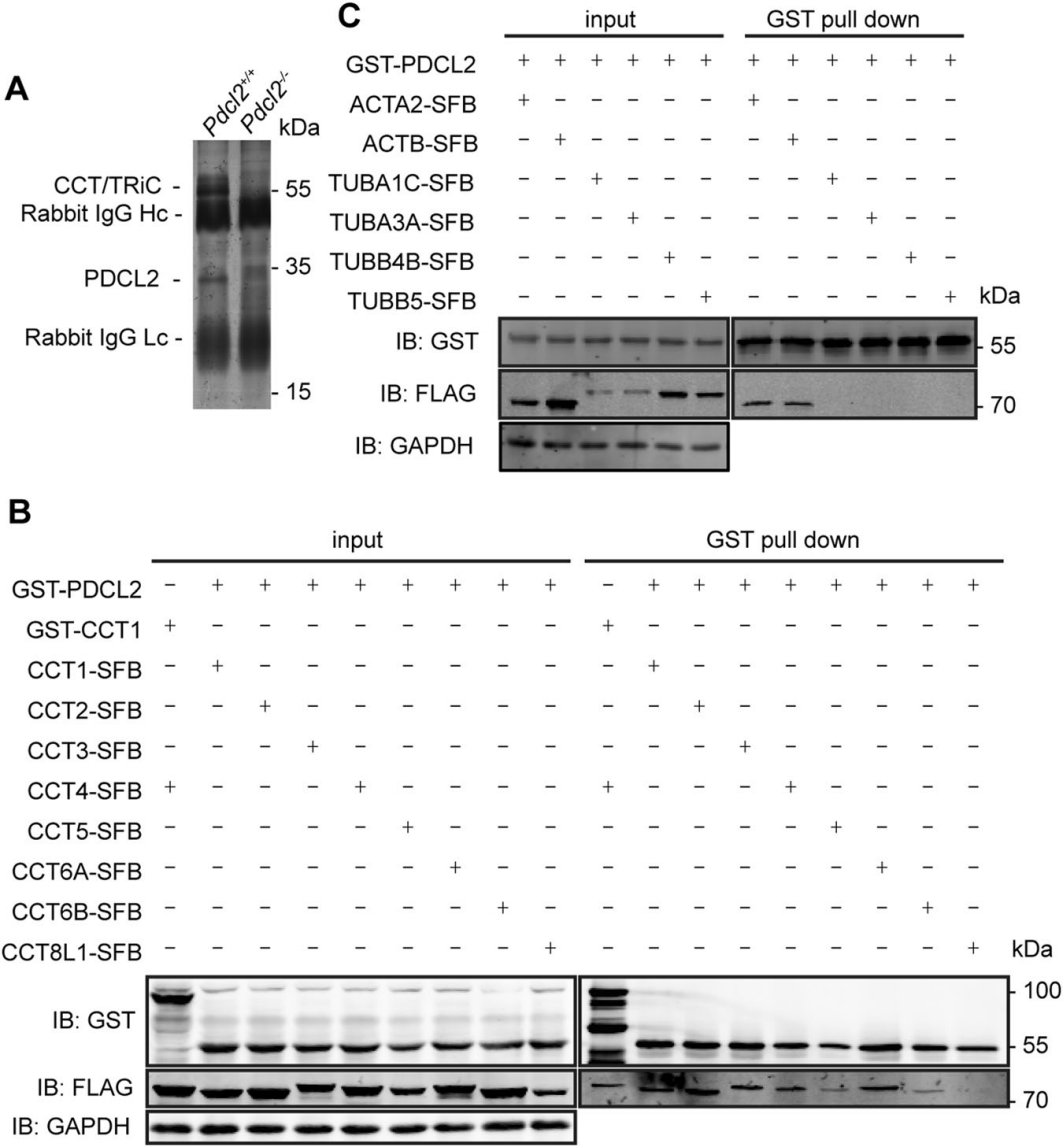
PDCL2 interacts with CCT and actin. **A** Coimmunoprecipitation of cleared extracts from testes of 11-week-old male mice with the PDCL2 antibody. Proteins were identified using mass spectrometry. Components of the CCT complex were the most abundant proteins interact with PDCL2. **B**, **C** GST pull down assay. Fusion proteins with N-terminal GST tag or C-terminal S tag-FLAG tag-SBP tag were over-expressed in 293T cells. Solubilized lysates were immobilized on Glutathione Sepharose 4B. Irrelevant proteins were washed away. Retained proteins were detected by immunoblotting with FLAG antibody. GAPDH was indicated as the loading control. CCT8L1 was used as a negative control. **B** Interaction between CCT components and PDCL2. **C** Interaction between actins and PDCL2.

In addition, sperm of *Pdcl2*^*−/−*^ mice were malformed without a regular hook-shaped head (Fig. 4C). On examining sperm morphology, we observed that nearly 50% of sperm from *Pdcl2*^*−/−*^ mice had round heads with no tail, whereas the remaining had more slender tails when compared with normal sperm (Fig. 4D). To examine the malformed structure of *Pdcl2*^*−/−*^ sperm head, we stained the sperm with the antibody of acrosome maker SP56. The result showed that *Pdcl2*^*−/−*^ sperm lacked a normal acrosomal structure, i.e., a typical crescent moon shape (Fig. 4E). We then examined the sperm structure using transmission electron microscopy (Fig. 4F). Sperm of *Pdcl2*^*−/−*^ mice failed to exhibit a normal mitochondrial distribution pattern. The mitochondria were clustered in a disordered manner. More white dots were observed in *Pdcl2*^*−/−*^ sperm heads than in those of *Pdcl2*^*+/+*^, indicating that nuclei were not properly condensed. Heterogeneous abnormalities were detected in *Pdcl2*^*−/−*^ sperm, including twined heads and tails, branched axonemes, and large vacuoles (Figs. 4F, 3–5).

*Pdcl2*^*−/−*^ mice had reduced sperm, which were immotile and malformed, indicating that these mice were suffering from oligo-astheno-teratozoospermia, and that was the underlying cause of infertility.

### PDCL2 Interacts with CCT and Actin

To elucidate the molecular function of PDCL2 in spermiogenesis, we used the PDCL2 antibody to pull down PDCL2 and PDCL2-associated protein(s) from testis protein extracts and identified the proteins by mass spectrometry. The most abundant proteins that interacted with PDCL2 were CCT components (Fig. 5A, Table S1). The GST pull-down assay further confirmed the interaction of CCT components with PDCL2 (Fig. 5B), in line with previous study performed in yeast [14–16, 33, 34]. PLP2 has been identified as a co-factor for actin folding by CCT [14–16]. We found that PDCL2 could also interact with actin in mammalian cells (Fig. 5C). Considering that the function of PDCL2 is conserved from yeast to mammals and transfection of mouse PDCL2 could rescue defects in PLP2 deficient yeast [3], PDCL2 might play a role in regulating actin protein folding during spermiogenesis in mice.

## Discussion

PDCL2 is a germline-specific protein. As expected, disruption of *Pdcl2* leads to reproductive malfunction in mice; however, this malfunction only occurred in male mice and not in females. This finding did not precisely meet our initial hypothesis, given that *Pdcl2* was detected in male spermiogenesis stages, as well as the meiotic stages of both sexes (Fig. 1C and Fig. S3C–F) [3]. In addition, PDCL2 has a conserved meiotic function during evolution. *Plp2*, the yeast orthologue of mouse *Pdcl2*, is essential for yeast to produce haploid cells, and the PLP2 defected phenotype can be rescued by overexpressing mouse PDCL2 in yeast [3]. Therefore, it was unexpected that successful meiosis would be observed in male or female *Pdcl2*^*−/−*^ mice. It can be speculated that PDCL2 no longer has any meiotic function in mice; however, this fails to explain the phenomena that mouse PDCL2 can rescue yeast meiotic defects.

Another possibility is that PDCL3, a paralog of PDCL2, compensates for the PDCL2 meiotic function. Considering that PDCL2 and PDCL3 exhibit a very high sequence similarity (Fig. S5A), and both can interact with CCT (Fig. 5A, B) [14], they may possess a similar function. In some cases of evolution, after gene duplication, the two daughter genes might mutate and change some expression control elements, resulting in distinct temporal and spatial expression patterns. Although they exhibit similar functions, both PDCL2 and PDCL3 are necessary and maintained in the genome [35]. PDCL2 and PDCL3 were separated and initially fixed in reptiles (Fig. S5B). PDCL2 expression is regulated by MYBL1 (A-MYB)-controlled RFX2 [36] and specifically expressed in the germline (Fig. 1 and Fig. S3) [3, 4], whereas PDCL3 was ubiquitously expressed. During spermatogenesis, during the pachytene stage of meiosis, PDCL2 starts to express, and PDCL3 starts to decay (Fig. S3C, D and Fig. S6A, B). It can be postulated that the remaining PDCL3 protein can function during meiosis. Additional information is required to demonstrate the relationship between PDCL2 and PDCL3.

Although the critical role of PDCL2 in spermiogenesis needs to be comprehensively clarified, we found that PDCL2 can interact with CCT and actin (Fig. 5), which strongly supports the role of PDCL2 for functional actin production. In fact, other members of subgroup II phosducin-like proteins (PhLP2) were found to modulate CCT function and assist or interfere with actin folding [14–16]. However, the details of this mechanism remain to be elucidated.

## Materials and methods

### Ethics statement

All experiments were conducted with protocols approved by The Institutional Animal Care and Use Committee at Sun Yat-sen University.

### Animals

Mice used in all experiments were C57BL/6. Mice were housed in specific pathogen-free (SPF) animal facility in Sun Yat-sen University. The Institutional Animal Care and Use Committee of Sun Yat-sen University, P.R.China approved all the experimental protocols concerning the handling of mice. *Pdcl2* knock-out mice were generated and tested following the previously described protocol [37].

We generated gRNAs expressing vector with gRNAs sequences as follows:

gRNA1: ttgtgcccctcactgggatc (AGG)

gRNA2: ggtgttgcgcttacagc (AGG)

100 ng/μL gRNA1, 100 ng/μL gRNA2, and 200 ng/μL Cas9 mRNAs were mixed and then 10 pL of the mixture was injected into the cytoplasm of each zygote. Mice were genotyped using the same primers as the T7E1 assay primers.

T7E1 assay primers are as follows:

T7E1-E1-F: 5′-GGACTCAGGGGTAAAAGGCG-3′,

T7E1-E1-R: 5′-TGACTAACAAAGAGAGACACTGCTAAAG-3′,

T7E1-E2-F: 5′-CTGAGTGGATGGGCAGTGTT-3′,

T7E1-E2-R: 5′-TGTGTCCTGTGAATCAAAGCA-3′.

Mutant mice of the first generation were mated with wild-type C57BL/6 for three generations trying to eliminate the uttermost off-target effect of the CRISPR/Cas9 system. The fourth generation of *Pdcl2*^*+/−*^ mice were mated to generate *Pdcl2*^*+/+*^, *Pdcl2*^*+/−*^, and *Pdcl2*^*−/−*^ mice. Mice of the same age from different groups were selected randomly to be analyzed.

### Semi-quantitative RT-PCR

RNA of different mouse tissues were extracted using the TRIzol reagent (Invitrogen, 15596–018). RNA samples were reverse transcripted using the RevertAid First Strand cDNA Synthesis Kit (Thermo Scientific, #K1621). Semi-quantitative RT-PCR conditions were as previously described [37]. Primers information were as follows:

*β-actin*-F: 5′-TTCTTTGCAGCTCCTTCGTTGCCG-3′,

*β-actin*-R: 5′-TGGATGGCTACGTACATGGCTGGG-3′,

*Pdcl2*-F: 5′-TGGCACAACTGAAAGAAGCAGA-3′,

*Pdcl2*-R: 5′-GCTTGAGATTTATCCCTCCACAT-3′.

### Generation of rabbit anti-PDCL2 polyclonal antibody

The entire protein coding sequence of mouse *Pdcl2* (NM_023508.7 CDS without stop codon) was cloned into pENTR/D-TOPO vector (Invitrogen) and recombined to pDEST15 (Invitrogen) using LR reaction (Invitrogen, 11791–020). The GST-PDCL2 fusion protein was expressed in Escherichia coli BL21 (DE3) and purified using Glutathione Sepharose 4B (GE Healthcare, 17-0756-01) according to the manufacturer’s instructions. Rabbits were immunized with the purified protein (Shanghai Institutes for Biological Sciences, CAS).

The entire protein coding sequence of mouse *Pdcl2* (NM_023508.7 CDS without stop codon) was cloned into pGEX-5X vector (GE Healthcare). The GST-PDCL2 fusion protein was expressed and purified as described above. The purified protein was used as an antigen to purify the rabbit serum using an antigen-coupled column generated with AminoLink Plus Coupling Resin (Pierce, 20501). Antibody specificity and sensitivity were tested (Fig. S2).

### Western blot analysis

Experiment procedures were described as previously [37]. Usages of primary antibodies were as follows:

rabbit anti-PDCL2 antibody (1: 1000),

mouse anti-GAPDH antibody (1: 8000; Proteintech group, 60004-1-Ig),

mouse anti-GST antibody (1:5000; Abmart, M20007),

rabbit anti-FLAG antibody (1:5000; Sigma-Aldrich, F7425)

Usages of secondary antibodies were as follows:

Goat anti-mouse IgG (1: 10,000; LI-COR, 926–32220),

goat anti-rabbit IgG (1: 10,000; LI-COR, 926–32211).

### Histological analysis

Bouin’s solution fixed mouse testes and epididymis were embedded in paraffin. Tissue-paraffin blocks were sectioned in 2 μm. Tissue slices were rehydrated sequentially and stained with hematoxylin and eosin.

### Immunofluorescent analysis of germ cells

Mouse germ cells were prepared and stained as previously described [37]. Usages of primary antibodies were as follows:

rabbit anti-PDCL2 antibody (1: 5000),

goat anti-PLZF antibody (1:500; R&D Systems, AF2944),

mouse anti-GATA4 antibody (1:100; Santa Cruz Biotechnology, sc-25310),

mouse anti-SYCP3 antibody (1:200; Santa Cruz Biotechnology, sc-74569),

mouse anti-sp56 antibody (1:1000; QED Bioscience, 55101).

Usages of secondary antibodies were as follows:

Alexa Fluor 488-conjugated donkey anti-goat IgG (1: 500, Molecular Probes, A-11055),

Alexa Fluor 488-conjugated donkey anti-mouse IgG (1: 500, Molecular Probes, A-21202),

Alexa Fluor 555-conjugated donkey anti-rabbit IgG (1: 500, Molecular Probes, A-31572).

### Immunofluorescent analysis of testes sections

Experiment procedures were as previously described [37] with modifications. Testes were fixed in PFA. Tissue-paraffin blocks were sectioned into 7 μm slices.

Usages of primary antibodies were as follows:

rabbit anti-PDCL2 antibody (1: 5000),

Usages of secondary antibodies were as follows:

Alexa Fluor 555-conjugated donkey anti-rabbit IgG (1: 500, Molecular Probes, A-31572).

### Sperm quantity and motility analysis

Sperm from cauda epididymis was analyzed as previously described [37].

### Transmission electron microscope analysis of sperm

Sperm samples were collected as previously described [37]. Samples were centrifuged and washed with PBS for three times at 500 × *g* for 2 min each time. Sperm samples were fixed by adding 1 mL glutaric dialdehyde and stored at 4 °C overnight. Samples were stained with uranyl acetate and lead citrate performed by Research Center of School of Life Sciences, Sun Yat-sen University. Images were captured with JEM1400 (JEOL).

### Analysis of apoptotic cells

Optimal cutting temperature compound (Sakura, 4583) embedded mouse testes and epididymis were cryosectioned into 10 μm slices. TUNEL assay was performed according to the manufacturer’s instruction (Roche, 11684795910).

### Analysis of germ cell ploidy types

Germ cell suspensions were prepared and stained with propidium iodide as previously described [37]. Flow cytometry analyses were performed using FACS Calibur (BD).

### Coimmunoprecipitation coupled with mass spectrometric identification (IP-MS)

Equally weighted mouse testes tissue of each group were homogenized in cold NETN-G buffer (40 mM Tris-HCl pH 8.0, 100 mM NaCl, 0.5% NP40, 1 mM EDTA pH 8.0, 10% glycerol) with 1:500 Cocktail (Sigma, P3840), 10 mM NaF, 0.2 mM PMSF, 1 mM DTT, 2 mM Na_4_P_2_O_7_ and 1 mM Na_3_VO_4_. The homogenates were Ultra-centrifuged at 24,000 rpm for 30 min at 4 °C. The sediment and upper lipid layers were discarded.

Preclear the lysate by incubating with 25 µL Rabbit IgG (Sangon Biotech, D110502) at 4 °C for an hour. Followed the incubation, 15 µL solid volume of precleared protein A/G agarose (Pierce, 20422) and 150 µL solid volume of precleared sefinose CL-6B (Sangon Biotech, SF004-L6B) were added to the mixture then incubated at 4 °C for 30 min. Centrifuge the mixture at 3000 rpm for 1 min at 4 °C (Eppendorf 5810R). The sediments were discarded. Centrifuge the supernatant at 20,000 × *g* for 30 min at 4 °C (Sigma 3–18 K centrifuge).

Binding the protein with PDCL2 antibody by adding 60 µL purified PDCL2 antibody to the supernatant and incubating at 4 °C for 3 h. After that, 15 µL solid volume of precleared protein A/G agarose were added to the mixture and incubated at 4 °C for 1.5 h. Centrifuge the mixture at 3000 rpm for 1 min at 4 °C (Eppendorf 5810R). The supernatant was discarded and the agarose beads were washed for three times with 1 mL NETN-G buffer each time.

Agarose samples were prepared to run SDS-PAGE. After electrophoresis, the gel was stained in coomassie brilliant blue buffer then cut out and sent to the Beijing Proteomics Research Center to perform the mass spectrometric identification.

### GST pull down assay

The full-length coding sequences of the target proteins were subcloned into the vector pDEST27 (Invitrogen) or pBabe-CMV-SFB, which generated fusion proteins with N-terminal GST tag or C-terminal S tag-FLAG tag-SBP tag. The vectors were transfected into 293T cells with polyethyleneimine (Sigma-Aldrich, 408727). Cells were lysed after 40 h of transfection. Cells were incubated with NETN-G buffer with 1: 100 cocktail and 10 mM NaF for 30 min at 4 °C. The homogenates were centrifuged at 15,000 rpm for 15 min at 4 °C. Supernatants were incubated with precleared Glutathione Sepharose 4B (GE Healthcare, 17-0756-01) at 4 °C for an hour. After incubation, beads were washed for three times with 0.5 mL NETN-G buffer each time. Samples were then prepared to run western blot analysis.

### Sequence analysis

Mouse PDCL2 and PDCL3 sequence (NP 075997.1 and NP 081126.2) were used to align with the online databases using the blastp suite (NCBI, protein-protein BLAST) to find out orthologues in other species. Multiple alignments were performed using MEGA7 and a phylogenetic tree was constructed. The evolutionary history was inferred using the Neighbor-Joining method. The evolutionary distances were computed using the Poisson correction method.

### Statistical analysis

All data are presented as mean ± S.D. SigmaPlot version 12.5 was used for analyzing data. The statistical significance of two groups were analyzed by using two-tailed *t-*test. The statistical significance of three groups were analyzed by using one way ANOVA. Normality test was performed using the Shapiro–Wilk method. Equal variance test was also performed. If there is a significant difference, Holm–Sidak method was used for all pairwise multiple comparisons. The data were considered significant when *p* < 0.05 (*), 0.01 (**) or 0.001 (***). Otherwise, it would be considered not significant (N.S.).

## Supplementary information

Supplementary Table

Supplementary Figure Legends

Western blots

Supplementary Figure 1

Supplementary Figure 2

Supplementary Figure 3

Supplementary Figure 4

Supplementary Figure 5

Supplementary Figure 6

## Data Availability

All data are available in the main text or the supplementary materials.
