# Supplementary material for: PDCL2 is essential for spermiogenesis and male fertility in mice
Source: Cell Death Discov. 2022 Oct 17;8:419. doi: 10.1038/s41420-022-01210-2 (PMC9576706; doi:10.1038/s41420-022-01210-2)
Supplement: Supplementary file 1 — Supplementary Table [file 41420_2022_1210_MOESM1_ESM.docx]

**Table S1 Proteins interact with PDCL2 were identified by IP-MS.**

| Gene name | Number of peptides in *Pdcl2^+/+^* group | Number of peptides in *Pdcl2^-/-^* group |
| --- | --- | --- |
| Cct8 | 24 | 0 |
| Cct2 | 19 | 0 |
| Cct5 | 17 | 0 |
| Cct7 | 18 | 2 |
| Cct3 | 18 | 4 |
| Tcp1 | 17 | 3 |
| Cct6b | 9 | 0 |
| Cct4 | 10 | 4 |
| Cct6a | 9 | 3 |
| Hspa1l | 5 | 0 |
| Tuba1c | 4 | 0 |
| Tuba3a | 4 | 0 |
