# Supplementary material for: PDCL2 is essential for spermiogenesis and male fertility in mice
Source: Cell Death Discov. 2022 Oct 17;8:419. doi: 10.1038/s41420-022-01210-2 (PMC9576706; doi:10.1038/s41420-022-01210-2)
Supplement: Supplementary file 2 — Supplementary Figure Legends [file 41420_2022_1210_MOESM2_ESM.docx]

**Supplementary Figure 1.** Normal people and teratozoospermia patients PDCL2 RNA expression level data extracted from the GEO dataset GDS2697. Data of this figure were represented as mean ± standard deviation. *t* test was performed. *, *p* < 0.05; **, *p* < 0.01; ***, *p* < 0.001.

**Supplementary Figure 2.** Western blot verification of PDCL2 antibody. Hela cells over-expressed PDCL2 with C-terminal S tag, FLAG tag and SBP tag fusion protein were transfected with 1, control siRNA or 2, Pdcl2 siRNA. Expression of the PDCL2 fusion protein was detected by immunoblotting with FLAG antibody or PDCL2 antibody. GAPDH was indicated as the loading control.

**Supplementary Figure 3.** PDCL2 RNA expression level data extracted from the GEO datasets. For the raw data, please refer to the GEO profiles and datasets accordingly. (A) Various normal human tissues, GDS3113. (B) Various mouse tissues, GDS3142. (C) Mouse male germ cells, GDS2390. (D) Mouse spermatogenesis time course, GDS606. Day post-partum. (E and F) Mouse ovary development time course, GDS2203 and GDS665. dpc, day post coitus. Data of this figure were represented as mean ± standard deviation. *, p < 0.05; **, p < 0.01; ***, p < 0.001. (A and B) One way ANOVA pairwise comparison. (C-F) One way ANOVA compare to the first column of the data.

**Supplementary Figure 4.** Representative pictures of TUNEL assay. (A) Testes cross sections. (B) Cauda epididymis cross sections. TUNEL signals were showed in green. Hoechst 33342 labeled cell nuclear was in blue. Bar, 100 µm.

**Supplementary Figure 5.** PDCL2 and PDCL3 are closely related. (A) BLAST of the PDCL2 and PDCL3 sequence. *, phosphorylation sites detected by mass spectrometry. (B) Phylogenetic relationships of the PDCL2 and PDCL3 proteins. This phylogenetic tree was constructed with the use of MEGA7. The evolutionary history was inferred using the Neighbor-Joining method. The evolutionary distances were computed using the Poisson correction method.

**Supplementary Figure 6.** PDCL3 RNA expression level data extracted from the GEO datasets. For the raw data, please refer to the GEO profiles and datasets accordingly. (A) Mouse male germ cells, GDS2390. (B) Mouse spermatogenesis time course, GDS605. Day post-partum. Data of this figure were represented as mean ± standard deviation. *, p < 0.05; **, p < 0.01; ***, p < 0.001.(A) One way ANOVA compare to the first column of the data. (B) One way ANOVA compare to the second column of the data.
