# Supplementary material for: PDCL2 is essential for spermiogenesis and male fertility in mice
Source: Cell Death Discov. 2022 Oct 17;8:419. doi: 10.1038/s41420-022-01210-2 (PMC9576706; doi:10.1038/s41420-022-01210-2)

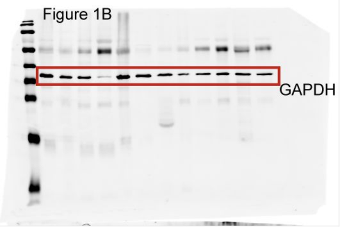
Figure 1B
GAPDH

Figure 1B

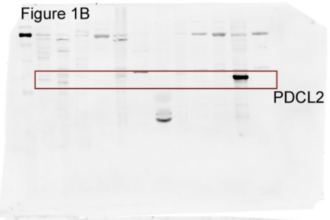

Figure 2B

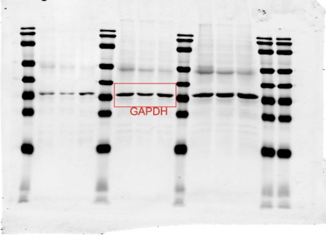

Figure 2B

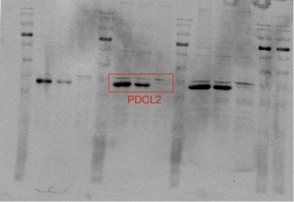

Figure 5A

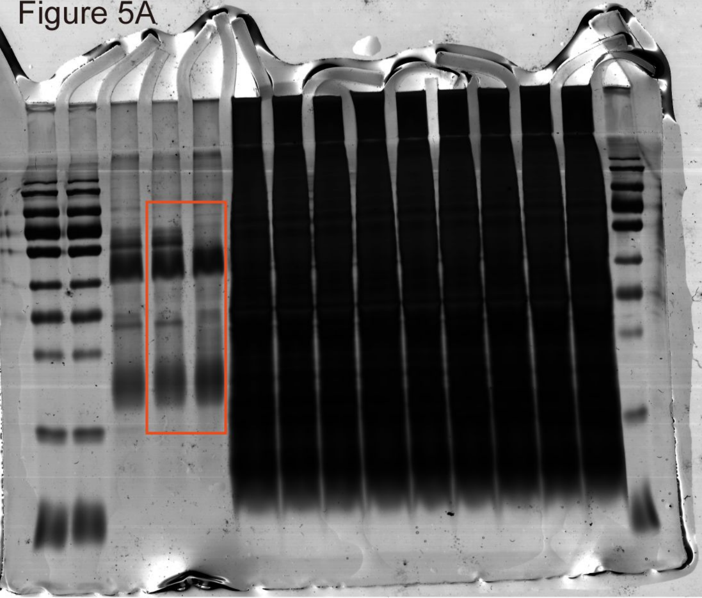

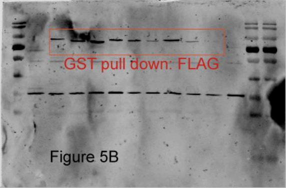

Figure 5B

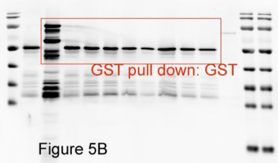

Figure 5B

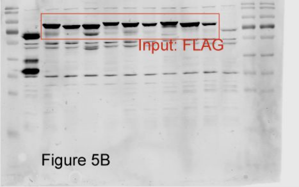
Input: FLAG
Figure 5B

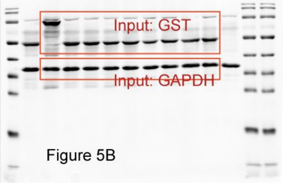

Figure 5B

Figure 5C

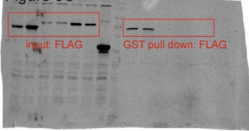

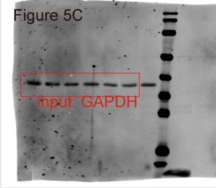
Figure 5C
input: GAPDH

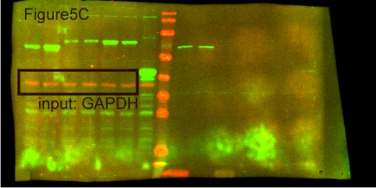
Figure5C
input: GAPDH

Figure 5C

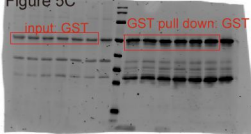

Supplementary Figure 2

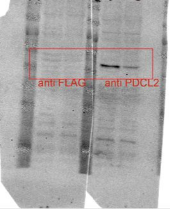

Supplementary Figure 2

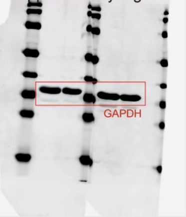

Supplement: Supplementary file 3 — Western blots [file 41420_2022_1210_MOESM3_ESM.pdf]
