# Supplementary figures and images for: PDCL2 is essential for spermiogenesis and male fertility in mice
Source: Cell Death Discov. 2022 Oct 17;8:419. doi: 10.1038/s41420-022-01210-2 (PMC9576706; doi:10.1038/s41420-022-01210-2)

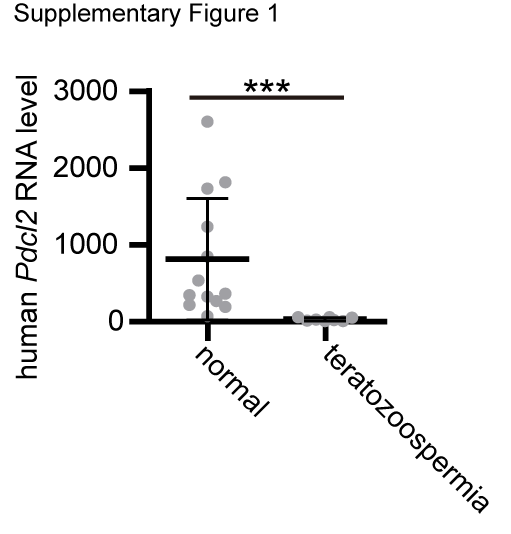

Supplement: Supplementary file 4 — Supplementary Figure 1 [file 41420_2022_1210_MOESM4_ESM.tif]

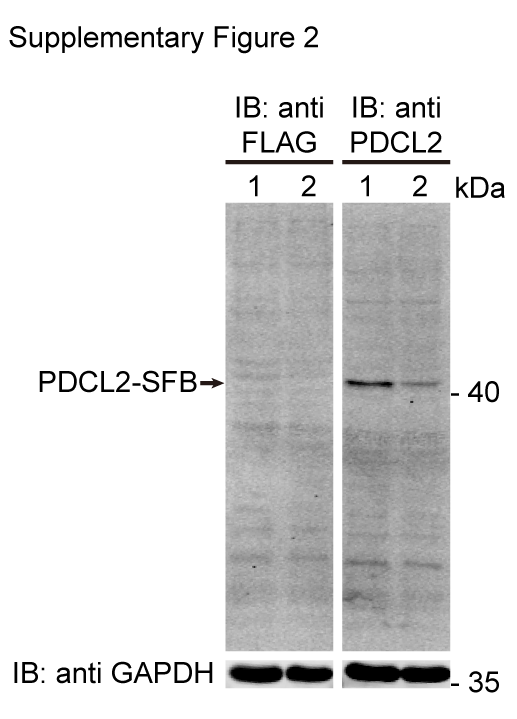

Supplement: Supplementary file 5 — Supplementary Figure 2 [file 41420_2022_1210_MOESM5_ESM.tif]

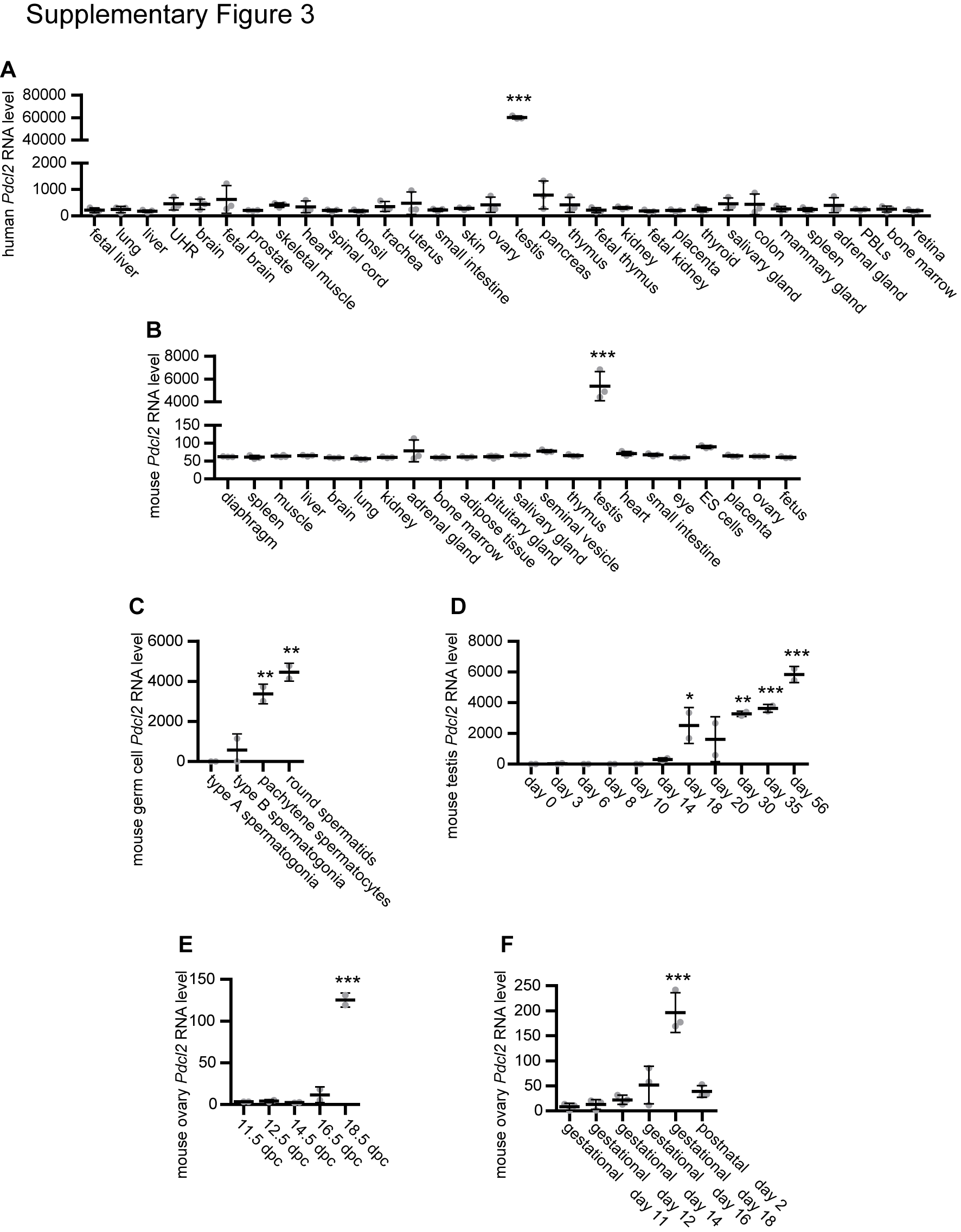

Supplement: Supplementary file 6 — Supplementary Figure 3 [file 41420_2022_1210_MOESM6_ESM.tif]

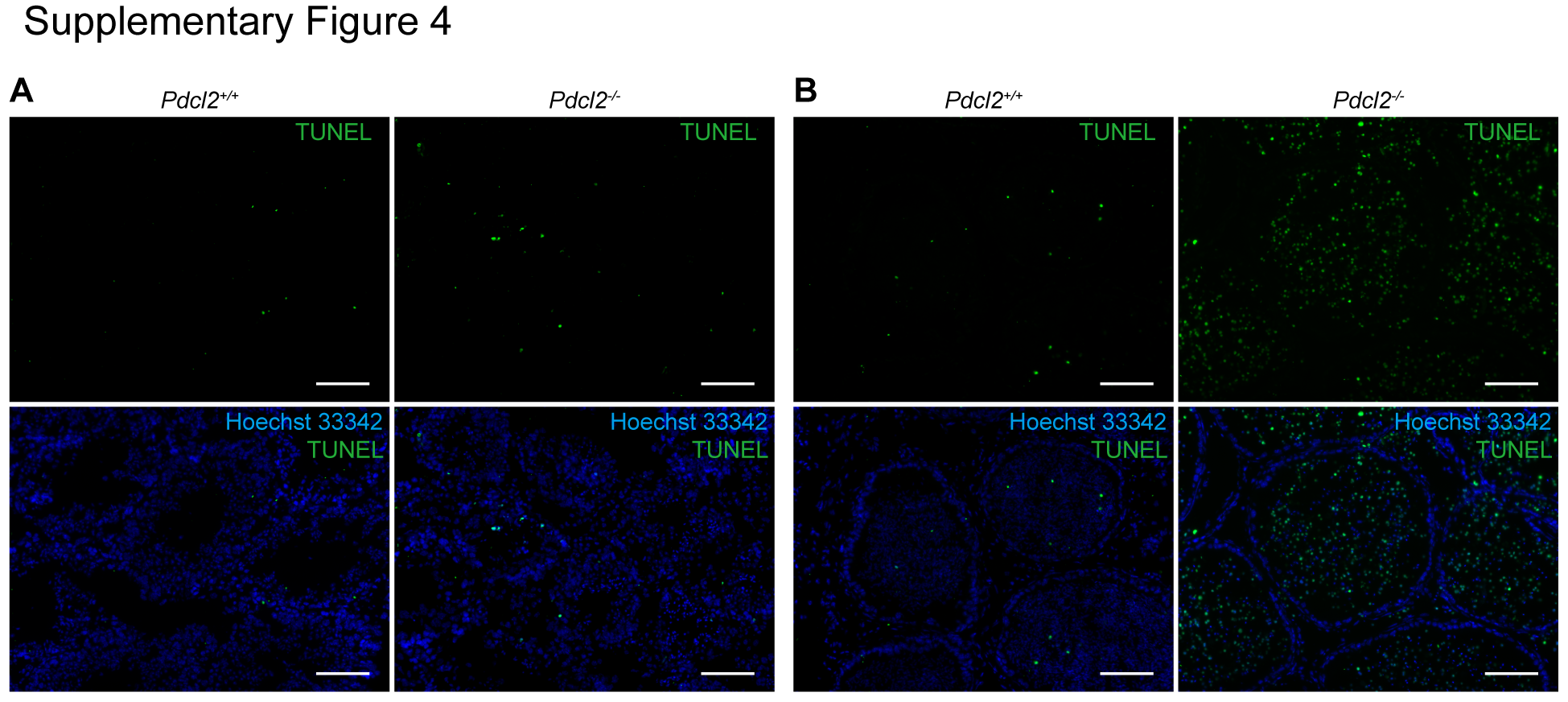

Supplement: Supplementary file 7 — Supplementary Figure 4 [file 41420_2022_1210_MOESM7_ESM.tif]

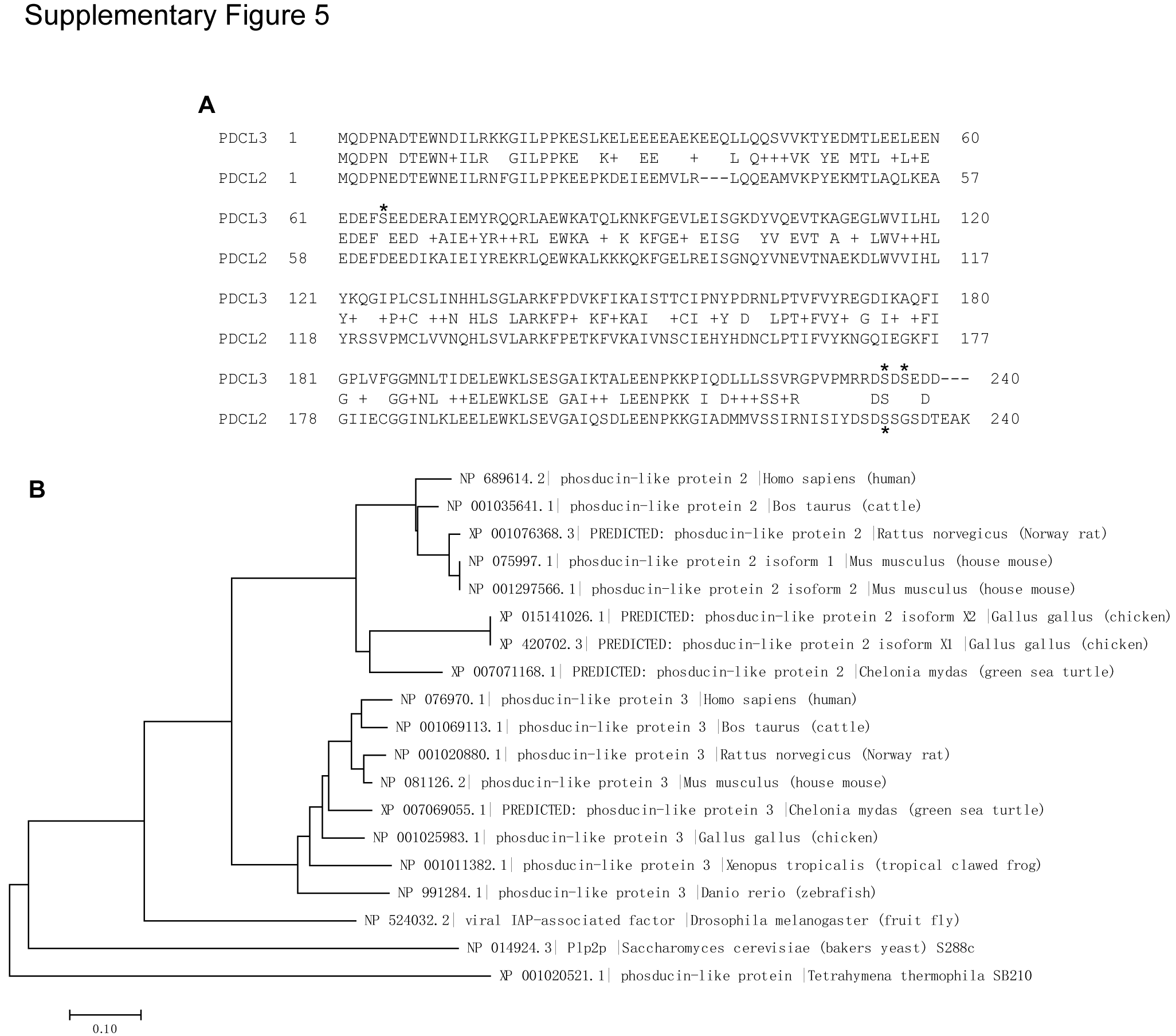

Supplement: Supplementary file 8 — Supplementary Figure 5 [file 41420_2022_1210_MOESM8_ESM.tif]

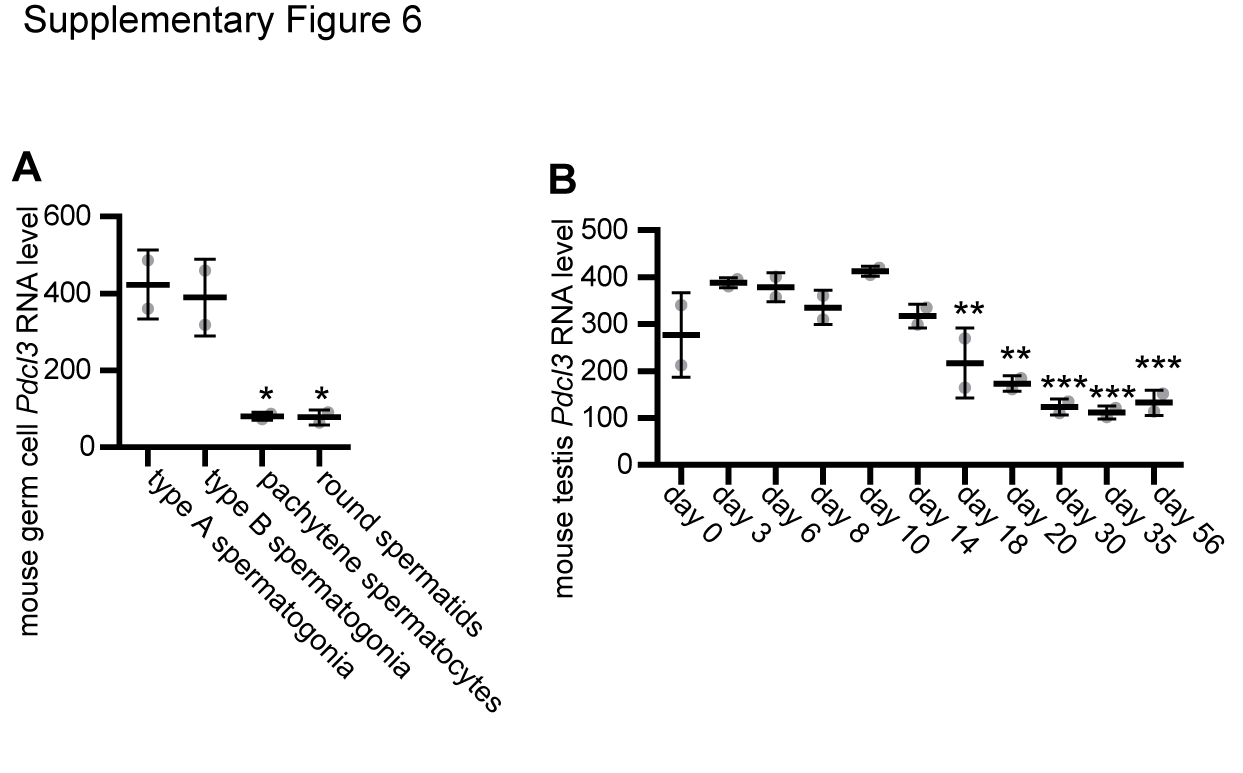

Supplement: Supplementary file 9 — Supplementary Figure 6 [file 41420_2022_1210_MOESM9_ESM.tif]
